# Supplementary material for: In silico assessment of 18S rDNA metabarcoding markers for the characterization of nematode communities
Source: PLoS One. 2024 Apr 5;19(4):e0298905. doi: 10.1371/journal.pone.0298905 (PMC10997105; doi:10.1371/journal.pone.0298905)
Supplement: Attachment — Submitted filename: response to reviewer comments.docx [file pone.0298905.s001.docx]

Dear editors,

We are glad of the positive comments on our manuscript entitled “In silico assessment of 18S rDNA metabarcoding markers for the characterization of nematode communities”. The manuscript has been modified following the reviewer suggestions. Specifically, we updated the figures following the Reviewer indications, we added new figures (weblogos) to show the mismatches in the primer region, and expanded the discussion on the trade-off between marker length and resolution. All the material needed to run analyses is now available online on Figshare.

We hope the manuscript will be now more suitable for publication with PlosOne

Please find below a letter where we detail the modifications of our manuscript

Yours sincerely

Gentile Francesco Ficetola, Alessia Guerrieri, Isabel Cantera, Aurelie Bonin

Response to the Journal Requirements, dr. Emily Chenette, and Iain Hrynaszkiewicz

1. Please ensure that your manuscript meets PLOS ONE's style requirements, including those for file naming. The PLOS ONE style templates can be found at

<https://journals.plos.org/plosone/s/file?id=wjVg/PLOSOne_formatting_sample_main_body.pdf> and

<https://journals.plos.org/plosone/s/file?id=ba62/PLOSOne_formatting_sample_title_authors_affiliations.pdf>

Done

*2. Please note that PLOS ONE has specific guidelines on code sharing for submissions in which author-generated code underpins the findings in the manuscript. In these cases, all author-generated code must be made available without restrictions upon publication of the work. Please review our guidelines at* [*https://journals.plos.org/plosone/s/materials-and-software-sharing#loc-sharing-code*](https://journals.plos.org/plosone/s/materials-and-software-sharing#loc-sharing-code) *and ensure that your code is shared in a way that follows best practice and facilitates reproducibility and reuse.*

*3. Note from Emily Chenette, Editor in Chief of PLOS ONE, and Iain Hrynaszkiewicz, Director of Open Research Solutions at PLOS: Did you know that depositing data in a repository is associated with up to a 25% citation advantage (https://doi.org/10.1371/journal.pone.0230416)? If you’ve not already done so, consider depositing your raw data in a repository to ensure your work is read, appreciated and cited by the largest possible audience. You’ll also earn an Accessible Data icon on your published paper if you deposit your data in any participating repository (https://plos.org/open-science/open-data/#accessible-data).*

The manuscript is based on data that are already accessible: the 18S-NemaBase (available at http://www.WormsEtAl.com/databases) and the GenBank database (version 249, available at https://ftp.ncbi.nlm.nih.gov/).

Furthermore, following the Editor’s suggestions, we provide the code and the data used to run analyses in figshare (10.6084/m9.figshare.24722199).

*4. Thank you for stating the following in the Acknowledgments Section of your manuscript:*

*“This study was funded by the European Research Council under the European Community’s Horizon 2020 Programme, Grant Agreement no. 772284 (IceCommunities).”*

*We note that you have provided funding information that is currently declared in your Funding Statement. However, funding information should not appear in the Acknowledgments section or other areas of your manuscript. We will only publish funding information present in the Funding Statement section of the online submission form.*

*Please remove any funding-related text from the manuscript and let us know how you would like to update your Funding Statement. Currently, your Funding Statement reads as follows:*

*“G.F.F., A.G., I.C. and A.B. have been funded by the European Research Council under the European Community’s Horizon 2020 Programme, Grant Agreement no. 772284 (IceCommunities).*

*The funders had no role in study design, data collection and analysis, decision to publish, or preparation of the manuscript.”*

*Please include your amended statements within your cover letter; we will change the online submission form on your behalf.*

Following this indication, we removed the funder information from the Acknowledgments section. The Funding Statement Section is fine.

*5. PLOS requires an ORCID iD for the corresponding author in Editorial Manager on papers submitted after December 6th, 2016. Please ensure that you have an ORCID iD and that it is validated in Editorial Manager. To do this, go to ‘Update my Information’ (in the upper left-hand corner of the main menu), and click on the Fetch/Validate link next to the ORCID field. This will take you to the ORCID site and allow you to create a new iD or authenticate a pre-existing iD in Editorial Manager*

Could you please change the name of the corresponding author?

The new corresponding author should be Isabel Cantera

ORCID: *0000-0003-3161-1878*

isa_cantera@hotmail.com

We tried to make the link on the submission system, but the website states that Isabel Cantera is already affiliated to this manuscript. Could you please check that ever tying is ok?

*6. We note that you have included the phrase “data not shown” in your manuscript. Unfortunately, this does not meet our data sharing requirements. PLOS does not permit references to inaccessible data. We require that authors provide all relevant data within the paper, Supporting Information files, or in an acceptable, public repository. Please add a citation to support this phrase or upload the data that corresponds with these findings to a stable repository (such as Figshare or Dryad) and provide and URLs, DOIs, or accession numbers that may be used to access these data. Or, if the data are not a core part of the research being presented in your study, we ask that you remove the phrase that refers to these data.*

We have rephrased this sentence, building on the specificity results from the in vitro analyses to strengthen our arguments and remove the reference to unpublished data.

*Response to the Editor’s comments

I got the recommendations and comments from an expert reviewer in the field. Both reviewers agree that the manuscript is technically sound and the data support the conclusions.
However, the lack of an explanation in the Methods, and Results and presentation was suggested by a reviewer, and I shared the comments. Therefore, I can invite you to submit a revised version of the manuscript that addresses the points raised by the reviewers.*

We modified the manuscript following the Reviewer suggestions, adding all the requested details.

Response to the Reviewer comments

*Reviewer #1: The paper addresses the crucial role of nematodes as keystone actors in various ecosystems and emphasizes the limitations posed by the complexity of morphological identification for monitoring their biodiversity on a broad scale. The adoption of DNA metabarcoding has emerged as a promising approach for assessing nematode biodiversity; however, the success of this method hinges on the utilization of universal primers with high taxonomic coverage and resolution. The authors undertake a comprehensive in-silico analysis of fourteen commonly used and one newly optimized primer for nematode metabarcoding, leveraging a recently available reference database. The evaluation encompasses key parameters such as taxonomic coverage, resolution, and specificity. The findings reveal that while most primers demonstrate excellent coverage, a subset exhibits limited coverage. Notably, all primers exhibit good taxonomic resolution, particularly at higher taxonomic levels like genera or families. The study underscores that species-level resolution tends to be higher for primers amplifying longer fragments. One notable observation is the lack of high specificity for nematodes across all primers, as they amplify a substantial number of other eukaryotes. This insight emphasizes the complexity in choosing markers appropriate for nematode metabarcoding, necessitating a trade-off between taxonomic resolution and amplified fragment length. In conclusion, the in-silico analyses presented in this paper provide valuable insights into selecting optimal primers based on study goals and the origin of DNA samples. This work represents a critical step in the design and optimization of metabarcoding studies aimed at assessing nematode diversity. The introduction effectively contextualizes the importance of nematodes in ecosystems, and the paper overall contributes to advancing our understanding of nematode biodiversity using molecular techniques.*

Many thanks for the positive comments

*However, I think a major flaw of this study is for comparing the primer specificity. The study mainly showed the primer specificity using specificity value. But I think which base on the primer has mismatching would be more important.*

Following this suggestion, we added as new analysis the weblogos, showing the mismatches between each base of the primers, and both target (nematode) and non-target taxa. These new figures have been added as figures 6-7. We expanded the methods (L 145-155), the results (L 189-191) and the discussion (L 207-211) to incorporate these new analyses

*Also, I think the trade-off between taxonomic resolution and the length of amplified fragments is a key observation. The paper should provide a more detailed discussion on the practical implications of this trade-off in the context of different research goals and objectives. Recommendations for researchers navigating this trade-off would be beneficial.*

Following this suggestion, we re-organized and expanded the discussion about the trade-off between taxonomic resolution and length, providing indication depending on study aim, type of substrate, and number of analyzed samples (costs-effectiveness) (L 275-286, 301-305)

*Comments on figures

As new Figure 1, It would be better to show a figure for the DNA regions in 18S with the primer regions.*

Following this suggestion, we added a new figure showing the primers within the 18S rDNA region (Figure 1).

*Figure 3: We acknowledge the difficulty in discerning coverage differences in Figure 3. To address this concern, we plan to enhance the visualization by adjusting the color scheme, possibly employing contrasting colors or patterns to highlight distinctions more effectively. This modification aims to provide a clearer representation of the coverage variations, ensuring that readers can easily interpret and compare the data.*

*Figure 4 and Others: In response to the suggestion of using different colors for the gene regions in Figure 4 and subsequent figures, we find this to be a valuable enhancement.*

Following these suggestions, we changed the color palettes for Fig. 2, Fig 4 and Fig. 5. We used the discrete rainbow scheme optimized for color-blind vision (<https://personal.sron.nl/~pault/#sec:qualitative>). We hope differences are now clearer.

*6. PLOS authors have the option to publish the peer review history of their article. If published, this will include your full peer review and any attached files.*

We agree to the publication of the peer review history of the paper.

Many thanks for the useful suggestions

Francesco Ficetola, Isabel Cantera, Alessia Guerrieri, Aurelie Bonin
